# Role of programmed death ligands in effective T-cell interactions in extranodal natural killer/T-cell lymphoma

**DOI:** 10.3892/ol.2014.2356

**Published:** 2014-07-17

**Authors:** LIJUAN HAN, FEIFEI LIU, RUPING LI, ZHAOMING LI, XINFENG CHEN, ZHIYUAN ZHOU, XUDONG ZHANG, TENGPENG HU, YI ZHANG, KEN YOUNG, SUKE SUN, JIANGUO WEN, MINGZHI ZHANG

**Affiliations:** 1 Department of Oncology, The First Affiliated Hospital of Zhengzhou University, Zhengzhou, Henan 450000, P.R. China; 2 Department of Sports Medicine, Research Center of Sports Medicine, Xiangya Hospital of Central South University, Changsha, Hunan 410008, P.R. China; 3 Biotherapy Center, The First Affiliated Hospital of Zhengzhou University, Zhengzhou, Henan 450000, P.R. China; 4 Department of Hematopathology, The University of Texas MD Anderson Cancer Center, Houston, TX 77230-1439, USA; 5 Institute of Clinical Medicine, The First Affiliated Hospital of Zhengzhou University, Zhengzhou, Henan 450000, P.R. China

**Keywords:** programmed death 1 ligand, B7 family, lymphoma, programmed death 2 ligand, programmed cell death 1, natural killer/T cell

## Abstract

Extranodal natural killer/T-cell lymphoma (ENKL) is marked by a profound cellular immune deficiency that may influence the capacity of T cells to extract an efficient antitumor immune response. It has been confirmed that the B7-CD28 pathway may promote tumor immune evasion by providing a negative regulatory signal. The current study analyzed the expression of programmed death 1 (PD-1)/programmed death ligand (PD-L) in ENKL cell lines and tissues. The functional studies were performed to analyze the functional activity of PD-L1 interacting with effective T cells in ENKL. PD-L1 and PD-L2 mRNA levels in ENKL cell lines were markedly upregulated compared with those in normal natural killer cells. The proteins constitutively expressed in the 30 ENKL specimens were significantly higher than in the 20 rhinitis specimens. In addition, PD-L1 and PD-L2 expression were found to closely correlate with certain clinical histopathological parameters. Furthermore, the count of PD-1^+^ tumor-infiltrating T lymphocytes was found to negatively correlate with the expression of PD-L1 and PD-L2. The PD-1 expression in the CD4^+^ and CD8^+^ T-cell subsets of 20 ENKL patients prior to therapy were significantly higher than that of the 10 healthy volunteers. In the functional studies, the cytokines (interleukin-2 and interferon-γ) secreted by CD8^+^ T cells were inhibited by PD-L1 expression in SNK-6 cells and this was restored with the presence of the PD-L1 blocking antibody. However no direct effect of PD-L1 was identified on CD8^+^ T-cell apoptosis and CD8^+^ T-cell cytotoxicity, as assessed by the proliferation of SNK-6 cells in the presence or absence of the neutralizing anti-PD-L1 antibody. The results of the current study revealed that PD-Ls and PD-1 are aberrantly expressed in ENKL and, furthermore, PD-L1 expression in SNK-6 cells was found to inhibit the activity of CD8^+^ T-cell cytokine secretion. This indicated that the PD-Ls may prevent effective antitumor immunity *in vivo* by interacting with tumor T cells, which provides important evidence to delineate the cellular immune deficiency mechanism in ENKL. Therefore, PD-1/PD-Ls are predicted to become novel targets for ENKL immunotherapy.

## Introduction

Extranodal natural killer/T-cell lymphoma (ENKL) is a rare subtype of non-Hodgkin lymphoma (NHL) and is characterized by distinct morphology, immunophenotype and biological behavior (1). According to the current World Health Organization (WHO) classification, extranodal natural killer/T-cell lymphoma is divided into ENKL and the aggressive natural killer cell leukemia (ANKL) (2). The tumor cells of ENKL derived from natural killer (NK) cells and, more rarely, T cells closely correlate with Epstein-Barr virus (EBV) infection (2,3). In addition, ENKL is estimated to account for 3–8% of malignant lymphomas in China and the incidence is much higher than that in Western countries (4). According to the original location, ENKL can be divided into two major subtypes; nasal and extranasal diseases. The nasal and paranasal lesions, including the upper aerodigestive tract, account for ≤80% of ENKLs (5). Currently, immunosuppression or immune disorders frequently occur in ENKL patients and ENKL is recognized as a highly aggressive neoplasm with poor therapeutic effects and prognosis. Thus, no uniform treatment regimen for ENKL has been determined (5), and the causes of the condition require investigation. We hypothesize that the inhibitory signals expressed in tumor cells inhibit the effective T-cell immune response, which aids the tumor cells to evade and destruct the immune surveillance and, ultimately, promote the pathogenesis and progression of ENKL.

The identification of the costimulatory molecules of the B7-CD28 superfamily has led to tremendous advancements in understanding the mechanism of T-cell immunity in numerous tumors. The programmed death ligand (PD-L) 1 and PD-L2 are two ligands of programmed death 1 (PD-1) and are members of the B7 immunoglobulin superfamily. PD-1, an immune inhibitory receptor, is a member of the CD28 family and, as a negative regulator, inhibits T-cell activity by conjunction with signaling through the T-cell receptor (6). In previous studies, the PD-1-PD-L signaling system has been demonstrated to be pivotal in immune tolerance, autoimmune diseases, chronic infections and inflammatory diseases, and has demonstrated a negative role in regulating the immune response (7,8). Notably, the PD-1/PD-L pathway is involved in tumor immune evasion of cancers, including melanoma, lung, esophageal, breast, renal and ovarian cancers, as well as hematopoietic malignancies (9). Iwamura *et al* (10) demonstrated that the small interfering RNA-mediated knockdown of PD-L1 or PD-L2 may enhance tumor-specific human T-cell effector functions, such as interferon (IFN)-γ production and antigen-specific cytotoxicity. However, a series of clinical trials concerning the systemic administration of therapeutic antibodies for blocking PD-1 or PD-L1 have shown a promising clinical effect in several solid tumors (11,12). PD-L1 and PD-L2 have an extensive expression pattern in NHL, including T- and B-cell lymphoma (13); however, the expression has not yet been characterized in ENKL. The current study addressed the role of the PD-Ls, particularly PD-L1, in effective T-cell interactions in ENKL. The results are likely to provide important evidence to delineate the cellular immune deficiency mechanism in ENKL and a potential strategy for immunotherapy against ENKL.

## Materials and methods

### Cell lines and peripheral blood mononuclear cell (PBMC) separation

The human ENKL SNK-6 and YTS cell lines were used. The SNK-6 cell line was a gift from Professor Norio Shimizu (Chiba University, Chiba, Japan) and the cells were cultured in RPMI-1640 (Beijing Solarbio Science and Technology Co., Ltd., Beijing, China) medium containing 2 mmol/l glutamine, 100 U/ml penicillin and 100 μg/ml streptomycin, supplemented with 1,000 U/ml interleukin (IL)-2 (Beijing SL Pharmaceutical Co., Ltd., Beijing, China) and 10% human AB serum provided by the Blood Center of Henan Province (Zhengzhou, China). The YTS cell line was a gift from Professor Scott Kaufmann (Mayo Medical Center, Rochester, MN, USA) and the cells were cultured in RPMI-1640, supplemented with 1% non-essential amino acids and 10% fetal calf serum (FCS; Hangzhou Sijiqing Biological Engineering Materials Co., Ltd, Hangzhou, China). The following cell lines were stored in a liquid nitrogen container at the Institute of Clinical Medicine of the First Affiliated Hospital of Zhengzhou University (Zhengzhou, China) and cultured in RPMI-1640 supplemented with 10% FCS: Human acute T-lymphoblastic leukemia Jurkat cell line (Shanghai Institute of Cellular Biology of Chinese Academy of Science, Shanghai, China); human cutaneous T-cell lymphoma Hut-78 cell line (gift from Professor Scott Kaufmann; Mayo Medical Center); anaplastic large cell lymphoma (ALCL) Karpas-299 cell line (Shanghai Institute of Cellular Biology of Chinese Academy of Science); diffuse large B-cell lymphoma LY-1 and LY-8 cell lines (Shanghai Institute of Cellular Biology of Chinese Academy of Science); and Burkitt lymphoma Raji and Ramos cell lines (Shanghai Institute of Cellular Biology of Chinese Academy of Science). All cell lines were cultured at 37°C in a 5% CO_2_ humidified atmosphere. The logarithmic growth phase cells were collected for experiments.

The blood samples obtained from 20 ENKL patients were collected at diagnosis prior to therapy and samples from six of the 20 patients were collected at efficacy evaluation (one, two and three cycles) during the chemotherapy. All patients provided written informed consent. The blood samples of 10 healthy volunteers (HVs) were provided by the Blood Bank of Henan Province (Zhengzhou, China). The PBMCs were isolated by density-gradient centrifugation using Ficoll-paque (Tianjin Biotechnology Development, Tianjin, China) and used immediately for flow cytometry (FCM) analysis. The study was approved by the First Affiliated Hospital of Zhengzhou University (Zhengzhou, China).

### Tissue samples

The specimens of 30 ENKL patients (21 males and nine females; mean age, 47 years; age range, 15–68 years) and 25 rhinitis patients (15 males and 10 females; mean age, 52 years; age range, 12–76 years) were obtained from the First Affiliated Hospital of Zhengzhou University (Zhengzhou, China) between 2010 and 2012. The lesions were all located in the nasal cavity and the pathological tissues were formalin-fixed and paraffin-embedded (FFPE). The diagnosis and classification were performed according to the 2008 WHO diagnosis and classification criteria (2). Clinical staging was conducted according to Ann Arbor stage [stage I (n=6), stage II (n=15), stage III (n=5) and stage IV (n=4)] (14). The specimens were selected according to the following criteria: Patients who had not received radiotherapy and chemotherapy; and availability of complete clinical and pathological data. The slices were reviewed by two senior pathologists.

### Fluorescence-based quantitative polymerase chain reaction (qPCR)

In total, 5×10^6^ cells in logarithmic growth phase were collected. The total RNA was extracted using a RNA extraction kit spin column method (Qiagen, Beijing, China) according to the manufacturer’s instructions. Finally, 60 μl RNA was collected and RNA purity (D260/D280) was 1.8–2.0, tested using an ultraviolet spectrophotometer [SMA4000; Merinton (Beijing) Instrument, Ltd., Beijing, China]. Subsequently, reverse transcription was conducted according to the RevertAid™ First Strand cDNA synthesis kit (Fermentas, Shenzhen, China) recommendations. In total, 20 μl cDNA was obtained and stored at −20°C until use. The following primers (synthesized by Sangon, Shanghai, China) were used for cDNA amplification system: Forward, CAT CTT ATT ATG CCT TGG TGT AGC A and reverse, GGA TTA CGT CTC CTC CAA ATG TG for PD-L1; forward, CAA CTT GGC TGC TTC ACA TTT T and reverse, TGT GGT GAC AGG TCT TTT TGT TGT for PD-L2; and forward, TGA CGT GGA CAT CCG CAA AG and reverse, CTG GAA GGT GGA CAG GG for β-actin. The amplification products were 146, 110 and 205 bp, respectively. qPCR was performed using the SYBR Green Mixture kit (CoWin Biotech Co., Ltd., Beijing, China) according to the manufacturer’s instructions. The data was normalized to the β-actin expression of NK cells and analyzed using the ABI 7500 Fast system (Applied Biosystems, Foster City, CA, USA). The qPCR was repeated three times.

### Immunohistochemistry (IHC)

The IHC streptavidin-peroxidase (SP) staining method was performed on 4 μm-thick FFPE tissue sections containing NKTL and rhinitis tissues. The sections were deparaffinized, dehydrated in xylene and graded ethanol solutions. The antigen retrieval was conducted in 0.01 mol/l citrate (pH 6.0) and the slides were incubated overnight with rabbit anti-human PD-L1 polyclonal antibody (1:120; Proteintech, Chicago, IL, USA), rabbit anti-human PD-L2 polyclonal antibody (1:150) and mouse anti-human PD-1 monoclonal antibody (mAb; 1:100) (both ZSGB-BIO, Beijing, China) and phosphate-buffered saline was used as a blank control. Incubation of the biotinylated secondary antibody with horseradish peroxidase (HRP) and 3,3′-diaminobenzidine chromogen (all from ZSGB-BIO, Beijing, China) was performed sequentially. Next, the slides were counterstained with hematoxylin and then covered with neutral balsam. The PD-1, PD-L1 and PD-L2 protein expression in the rhinitis tissue served as controls.

### IHC scoring

PD-L1, PD-L2 and PD-1 expression were evaluated as staining at the cell membrane and cytoplasm. Positive staining for PD-L1 and PD-L2 was determined by staining intensity and the percentage of positive cells was determined according to the methods used in tumors (12,13,15). The following staining intensity grading was used: 0, no staining; 1, faint yellow; 2, claybank; and 3, sepia. The percentage of the positive tumor cells was scored as follows: 1, <10%; 2, 10–50%; 3, >50%. The positive cases were then classified according to the product of the staining intensity and positive tumor cell values as follows: Positive, >3 points; and negative, <3 points. The mean count of PD-1 positive cells of the 30 cases was used as the threshold and the cases were divided into high PD-1^+^ tumor-infiltrating T lymphocytes (TILs) and low PD-1^+^ TIL groups according to the threshold.

### Magnetic-activated cell sorting (MACS)

The PBMCs were isolated from the HVs using the previously described methods. The MACS was then conducted by a standard method. CD8^+^ T cells were separated using CD8 and CD56 microbeads (BD Biosciences, Heidelberg, Germany) for NKs. The purity of these cells was measured by FCM.

### FCM

FCM was performed by a standard method and the results were acquired using a FACSCanto II cytofluorimeter (BD Biosciences) and analyzed using BD CellQuest Pro software (BD Biosciences). The following antibodies were used to measure the expression of PD-1 in the PBMCs: Mouse monoclonal anti-human-CD3-peridinin (Percp) (Catalogue number: 347344, clone: SK7), monoclonal mouse anti-human-CD4-fluorescein isothiocyanate (FITC) (cat. no.: 340962, clone: SK3), monoclonal mouse anti-human-CD8-allophycocyanin-cyanine 7 (Cy7) (cat. no.: 557760, clone: RPA-T8) and monoclonal mouse anti-human-PD-1-phycoerythrin (PE)-Cy7 (cat. no.: 561272, clone: EH12.1). The mAb was used to measure the purity of CD8^+^ T and NK cells separated by MACS, including monoclonal mouse anti-human-CD8-Percp (cat. no.: 341004, clone: SK7), monoclonal mouse anti-human-CD3-PE-Cy7 (cat. no.: 557749, clone: SP34-2) and monoclonal mouse anti-human-CD56-FITC (cat. no.: 340410, clone: NCAM16.2). The mouse IgG-FITC isotype control antibodies were also used. The antibodies used in FCM were all purchased from BD Pharmingen (San Diego, CA, USA).

### Enzyme-linked immunosorbent assays (ELISA)

T-helper type 1 (Th1) cytokines (IL-2 and IFN-γ) that had secreted into the serum of the 20 ENKL patients and 10 HVs were detected by Quantikine HS ELISA kits (R&D Systems, Minneapolis, MN, USA). Blood samples were collected and, following centrifugation of the blood samples at 1,100 × g for 10 min, the serum was obtained. The standard and blank wells were placed separately in the coated ELISA plates and the test samples were diluted with sample diluent (R&D Systems) and added to the test sample wells. Following the addition of the HRP-conjugate reagent (with the exception of the blank well), the samples were incubated at 37°C in a humidified atmosphere of 5% CO_2_ for 72 h and washed five times with scrubbing solution (R&D Systems) subsequently the chromogen solutions A and B were added. Following five times washing of the wells with scrubbing solution, the stop solution was added to terminate the reaction. The blank well was taken as zero and the absorbance optical density values were read at 450 nm within 15 min. The concentrations of IL-2 and IFN-γ were determined by standard curves. All experiments were repeated three times.

### Functional experiments

To delineate the role of the PD-Ls in tumor T-cell interactions in ENKL, coculture experiments were performed by simulating the tumor microenvironment *in vivo*. The experiments were divided into the following groups: i) SNK-6/CD8^+^ T-cell control group; ii) SNK-6 and activated CD8^+^ T-cell group; and iii) SNK-6, activated CD8^+^ T-cell and anti-PD-L1 antibody group. The purified allogeneic CD8^+^ T cells were then plated in 24-well plates at a density of 6×10^6^ cells/well, and activated with phytohemagglutinin (PHA; 2 μg/ml, Sigma-Aldrich, St. Louis, MO, USA) for 48 h. Next, the allogeneic CD8^+^ T cells were cocultured with 6×10^5^ SNK-6 cells stained with 5 mmol/l carboxyfluorescein succinimidyl ester (CFSE; BD Pharmingen) in the presence of the anti-PD-L1 antibody (6 μg/ml; MIH1 clone; eBioscience, Inc., San Diego, CA, USA) or the mouse IgG1 isotype control mAbs. The ratio of effective and target cells was 10:1. For the measurement of the SNK-6 cell proliferation, the SNK-6 cells were used as the control group. The cells in each group were harvested at 0, 24, 48 and 72 h and analyzed by FCM gating CFSE^+^ events. Following the coculture of the SNK-6 and CD8^+^ T cells for 72 h, the supernatants were removed to measure the Th1 cytokine (IL-2 and IFN-γ) secretion by ELISA, of which the SNK-6 cells were used as the control group. CD8^+^ T-cell apoptosis was analyzed at 72 h by FCM gating Annexin V^+^ and 7-aminoactinomycin D (AAD)^+^ (Annexin-V-PE-A and 7-AAD-Percp-cy5-5-A; BD Pharmingen) cells, and the activated CD8^+^ T cells were simultaneously used as the control group.

### Statistical analysis

SPSS 17.0 software (SPSS, Inc., Chicago, IL, USA) was used for statistical analysis. Data are presented as the mean ± standard deviation. Spearman’s rank correlation test was used to examine the correlation between PD-L1 and PD-L2 and the clinicopathological parameters. The χ^2^ test was used to analyze the positive expression rate, and Student’s t-test and one-way analysis of variance were used to analyze the differences among the groups. For all comparisons, P<0.05 was considered to indicate a statistically significant difference.

## Results

### mRNA expression of PD-L1 and PD-L2 in NHL cell lines

**Figure 1:**
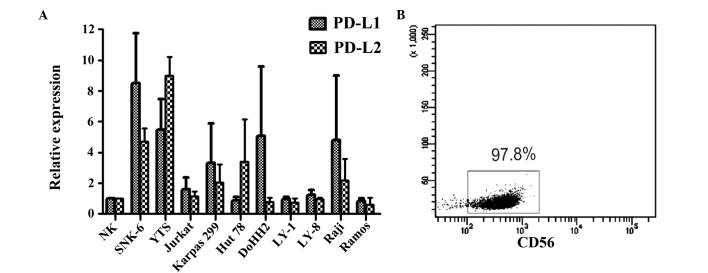
(A) The mRNA expression of PD-L1 and PD-L2 in extranodal NK/T-cell lymphoma and other non-Hodgkin lymphoma cell lines by fluorescence-based quantitative polymerase chain reaction. The data were normalized to the β-actin expression of NK cells. (B) The purity of NK cells separated by magnetic-activated cell sorting was 97.8%. PD-L, programmed death ligand; NK, natural killer.

The levels of PD-L mRNA in ENKL and other NHL cell lines were detected by qPCR. The expression of PD-L1 and PD-L2 in SNK-6 and YTS cells was significantly elevated compared with that in the normal NK cells (P<0.05; Fig. 1A). In addition, PD-L1 and PD-L2 were expressed in the T- and B-cell lymphoma cell lines. However, the expression of PD-L1 and PD-L2 mRNA in the T-cell lymphoma cell lines was higher than that in the B-cell lymphoma cell lines (Fig. 1A). The purity of NK cells separated by MACS was 97.8% (Fig. 1B). In addition, the expression of PD-1 was not detected in the ENKL cell lines, SNK-6 or YTS (data not shown).

### Expression of PD-L1, PD-L2 and PD-1 proteins, and correlation between PD-L1 and PD-L2 and clinical pathological parameters of ENKL

**Figure 2:**
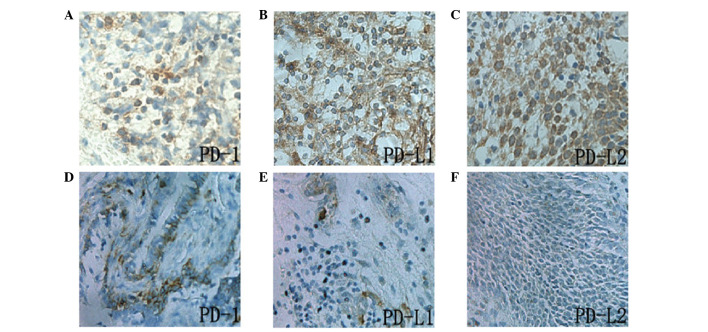
Representative immunohistochemical streptavidin-peroxidase staining in extranodal natural killer/T-cell lymphoma (upper row) and rhinitis tissues (lower row). The positive cases of (A and D) programmed death 1, (B and E) PD-L1 and (C and F) PD-L2 (magnification, ×200). PD-L, programmed death ligand.

PD-L1, PD-L2 and PD-1 proteins were all located in the cytoplasm and cell membrane, and the expression of PD-L1 and PD-L2 was detected in tumor and stromal cells. However, PD-1 was predominantly expressed in the tumor stromal lymphocytes (Fig. 2). The positive expression of PD-L1 and PD-L2 in ENKL tumor cells was 60.0 and 63.3%, respectively, which was significantly increased compared with that in the rhinitis tissues (16.0 and 12%; P<0.05). The count of PD-1^+^ TILs in the 30 ENKL cases (range, 0–18; mean, 7.56) was significantly increased in contrast to that in the rhinitis tissues (range, 0–9; mean, 1.52) (P<0.05). The mean value of 7.56 was used as the threshold and, according to this, the 30 ENKL cases were divided into a PD-1^+^ TIL high-density group (16 cases) and low-density group (14 cases). The expression of PD-L1 and PD-L2 was found to inversely correlate with PD-1^+^ TILs. Furthermore, the expression of PD-L1 and PD-L2 was found to positively correlate with tumor stage. PD-L1 but not PD-L2 expression was also found to positively correlate with the international prognostic index (IPI) and lactate dehydrogenase (LDH) and Ki-67 levels. However, no correlation was identified between PD-L1 and PD-L2 expression and the other clinical histopathological parameters (Table I).

### PD-1 expression in CD4^+^ and CD8^+^ T-cell subsets prior to therapy, and PD-1 expression variation tendency with chemotherapy

**Figure 3:**
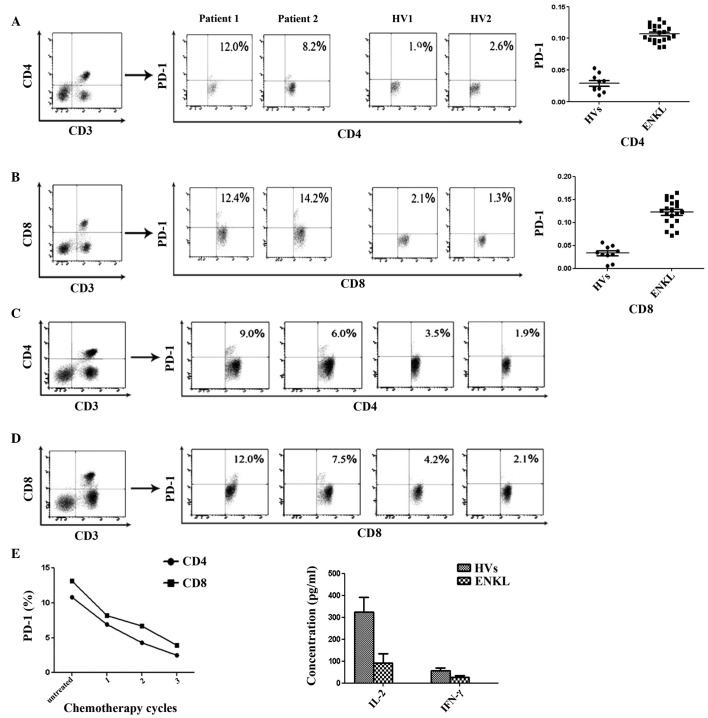
PD-1 expression in (A) CD4^+^ and (B) CD8^+^ T-cell subsets in 20 ENKL patients was significantly increased compared with that in 10 HVs (P<0.05). Representative PD-1 expression in (C) CD4^+^ and (D) CD8^+^ T-cell subsets in six ENKL patients was (E) downregulated with chemotherapy. (F) T-helper cell type 1 cytokine (IL-2 and IFN-γ) mean production levels in the serum of 20 ENKL patients were significantly lower than those in 10 HVs (P<0.05). PD1, programmed death 1; ENKL, extranodal natural killer/T-cell lymphoma; HVs, healthy volunteers; IL-2 interleukin 2; IFN-γ, interferon γ.

By applying morphological parameters and multicolor FCM, PD-1 expression in CD4^+^ (Fig. 3A) and CD8^+^ (Fig. 3B) T-cell subsets in 20 ENKL patients was significantly increased compared with that in the 10 HVs (P<0.05). Notably, PD-1 expression in the CD4^+^ (Fig. 3C) and CD8^+^ (Fig. 3D) T-cell subsets was downregulated with chemotherapy (Fig. 3E).

### Mean production levels of Th1 cytokines (IL-2 and IFN-γ) in the serum of 20 ENKL patients

The concentration of Th1 cytokines (IL-2 and IFN-γ) was obtained by standard curve. The mean production levels of IL-2 and IFN-γ in the serum of 20 ENKL patients were significantly lower than that of the 10 HVs (Fig. 3F; P<0.05).

### Functional significance of PD-L1 expression for purified allogeneic CD8^+^ T-cell interactions

**Figure 4:**
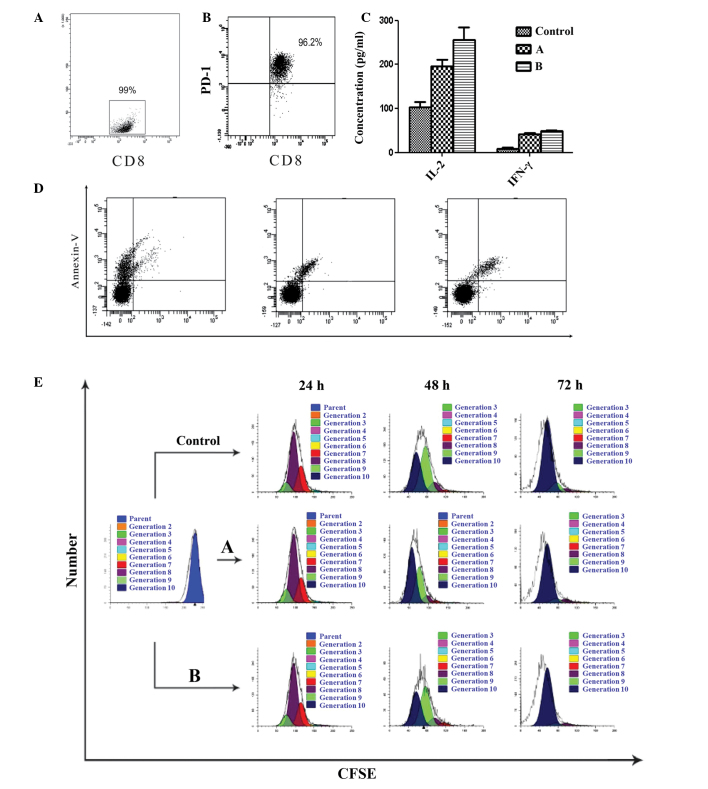
(A) Purity of CD8^+^ T cells separated by magnetic-activated cell sorting was 99%. (B) Purity of CD8^+^ PD-1^+^ T cells was 96.2% following the stimulation of allogeneic CD8^+^ T cells with phytohemagglutinin for 48 h. (C) SNK-6 cells were used as the control group and, following the coculture of SNK-6 cells and CD8^+^ T cells for 72 h, a significant inhibitory effect of PD-L1 on allogeneic CD8^+^ T-helper type 1 cytokine (IL-2 and IFN-γ) secretion was observed; (A and B) P<0.05. (D) CD8^+^ T-cell apoptosis in groups A and B was not altered significantly compared with activated CD8^+^ T cells at 72 h (P>0.05). (E) SNK-6 cells were used as the control group and cells harvested at 0, 24, 48 and 72 h were analyzed by flow cytometry gating CFSE^+^ events. The proliferation index was not significantly different among the groups (P>0.05). PD-1, programme death 1; PD-L. programmed death ligand; IL-2 interleukin 2; IFN-γ, interferon γ; CFSE, carboxy-fluorescein succinimidyl ester.

As PD-1 expression was elevated in the TILs of the ENKL tissues, and PD-L1 and PD-L2 expression was elevated in the tumor cells and cell lines, coculture experiments of the SNK-6 cells and purified allogeneic CD8^+^ T cells (effector:target ratio, 10:1) were established by simulating the tumor microenvironment *in vivo*. The purity of CD8^+^ T cells separated by MACS was 99% (Fig. 4A). In the functional studies, PD-1 expression was characterized in the allogeneic CD8^+^ T cells stimulated with PHA for 48 h, and the percentage of CD8^+^ PD-1^+^ T cells was 96.2% (Fig. 4B). A significant inhibitory effect of PD-L1 was identified for cytokine secretion (IL-2 and IFN-γ) in the allogeneic CD8^+^ T-cell subset, and this inhibitory effect was restored with the PD-L1 blocking antibody (P<0.05; Fig. 4C). The CD8^+^ T-cell apoptosis in groups A and B was not altered significantly compared with that of activated CD8^+^ T cells at 72 h (P>0.05; Fig. 4D). The SNK-6 cell proliferation was detected using CD8^+^ T-cell cytotoxic activity, and the proliferation index was not altered significantly (P>0.05; Fig. 4E).

## Discussion

ENKL is characterized by a highly aggressive clinical course, poor prognosis and is analogous to ANKL (16). Generally, ENKL patients have immunosuppression or immune disorders and, therefore, we suspect that the expression of immune inhibitory molecules in ENKL tumor cells impedes or paralyzes the antitumoral immune responses. It was indicated that using tumor antigen-specific CTLs against tumor cells is likely to have broad application prospects in the treatment of malignancies. However, the functional activity of tumor-specific T cells is likely to be attenuated by its receptor (CTLA-4 and PD-1) interactions with its ligands, which transfer negative regulation signals and ultimately prevent an effective antitumor immune response (10). Thus, the manner in which to improve tumor cell immunogenicity or interfere with the biological signal for immune evasion is likely to be via hotspots in ENKL immunotherapy studies.

The B7 immunoglobulin superfamily are the sole costimulatory molecules which deliver regulatory signals from the antigen-presenting cells (APCs) to the T cells. At present, the B7 family comprises eight members: CD80 (B7-1), CD86 (B7-2), CD274 (PD-L1 or B7-H1), CD273 (PD-L2 or B7-DC), CD275 (B7-H2 or ICOS-L), CD276 (B7-H3), B7-H4 (B7S1 or B7x) and B7-H6 (17). PD-L1 is predominantly expressed in activated T and B cells, APCs, (dendritic cells) DCs, macrophages and human tumor cells (16). PD-L2 is not only expressed in DCs and macrophages, but also in immunocytes, including Th2. The gene structure, gene sequence and function of PD-L2 are similar to that of PD-L1 (18), and the extracellular region of PD-1 is 28% identical to that of CTLA-4 (19). In addition, PD-1 is expressed in activated T and B lymphocytes, myeloid cells and thymocytes (16).

PD-L1 and PD-L2, but particularly PD-L1, are extensively expressed in entity tumors and hematopoietic malignancies (9). Although PD-L1 and PD-L2 molecules share 34% identity of amino acids, their expression has been suggested to be differentially regulated (20). Furthermore, PD-L1 exhibits a more extensive expression pattern and higher expression intensity than PD-L2 in Hodgkin’s lymphoma (HL) and NHL (21–23). However, the expression of PD-1, PD-L1 and PD-L2 in ENKL has never been shown. The current study detected the expression and distribution of PD-1, PD-L1 and PD-L2 in ENKL. The results showed that PD-L1 and PD-L2 are aberrantly expressed at a high level in the cell lines compared with normal NK cells. In addition, PD-L1 and PD-L2 were found to simultaneously exhibit higher expression levels in T-cell lymphoma than B-cell lymphoma. By contrast, the ENKL cell lines, SNK-6 and YTS, were found to lack expression of PD-1. In the IHC analysis, the PD-L1 and PD-L2 proteins were found to be located in the cytoplasm and cell membrane of tumor and stromal cells. However, PD-1 was predominantly expressed in tumor stromal lymphocytes. PD-L1 and PD-L2 expression in ENKL tumor cells was significantly increased in contrast to that in rhinitis tissues. In human pancreatic cancer, tumor PD-L1 but not PD-L2 expression showed a poorer postoperative prognosis and PD-L1 expression was found to inversely correlate with tumor antigen-specific CD8^+^ T cells (24). In human esophageal cancer, no correlation was identified between PD-L1 expression and TILs (25). However, no correlation was identified between PD-1 and PD-L1 expression and the stage of disease or LDH in chronic lymphocytic leukemia (26). The results of the current study found that PD-L1 and PD-L2 expression in tumor cells positively correlates with clinical stage. In addition, PD-L1 but not PD-L2 expression was found to positively correlate with LDH and Ki-67 levels, as well as negatively correlate with IPI score. However, no significant correlation was identified between PD-L1 and PD-L2 expression, and the other clinical histopathological parameters. Notably, Greene *et al* (27) using EBV-transformed B cells demonstrated that the EBV-encoded latent membrane protein 1 through activator protein 1 and JAK-STAT signaling can upregulate PD-L1 expression. The aberrant signaling through EBV-encoded gene products provides alternative mechanisms to promote PD-L1 expression in EBV-positive classical Hodgkin lymphoma and post-transplant lymphoproliferative disorders (13). However, the results of the current study showed no correlation between EBV-encoded small RNA and PD-L1 and PD-L2 expression in ENKL. However, the reason for this may be due to the limited number of cases. In the present study, PD-L1 and PD-L2 expression were found to negatively correlate with PD-1^+^ TILs. The results of the current study markedly indicated that ENKL tumor cells inhibit TIL activity or promote TIL apoptosis, ultimately promoting immune evasion via the PD-1/PD-L pathway. The Th1 cytokine (IL-2 and IFN-γ) levels were also detected in the serum and found to be significantly decreased. In addition, the immune inhibitory receptor PD-1 expression in CD4^+^ and CD8^+^ T cells was significantly upregulated in the 20 ENKL patients in contrast to that in the 10 HVs. These observations not only indicated that the immune function in ENKL patients is suppressed, but were also in favor of the theory that cellular immunity deficiency frequently occurs in cancer patients. PD-1 has been identified as a prognostic risk factor in follicular lymphoma and a marker of angioimmunoblastic T-cell lymphoma (28,29). However, the current study found that PD-1 expression levels are attenuated with chemotherapy. This phenomenon may be explained by the robust increase in the PD-1 expression of T lymphocytes in ENKL patients when the cells are activated by specific antigens. In addition, we suspect that chemotherapy may alter the tumor antigen-specific response.

PD-L1 has been proposed as a bridge to connect the innate and adaptive immunity in combating leukemia (30). In ovarian cancer, the lysis of tumor cells with PD-L1 overexpression in CTLs is attenuated, however, when PD-L1 is silenced, the lysis is promoted. In mouse models of ovarian cancer following peritoneal dissemination, PD-L1 depletion has been found to inhibit tumor growth and prolong survival (31). Andorsky *et al* (21) established a coculture system of ALCL cell lines and allogeneic T cells, and it was observed that T-cell proliferation and IFN-γ secretion was increased in the presence of the anti-PD-L1 blocking antibody. In conclusion, the activity of CTL was inhibited by tumor cells via the PD-L1/PD-1 negative regulation pathway. However, antitumor immune response was restored using a recombinant soluble PD-L1 or blocking antibodies to interfere with the PD-L1/PD-1 pathway in specific types of tumors. The current study simulated the *in vivo* tumor environment and CD8^+^ T cells were activated as effective cells by incubation with PHA for 48 h, and PD-1 expression was significantly elevated compared with the unactivated CD8^+^ T cells. Subsequently, SNK-6 cells and purified activated CD8^+^ T cells were cocultured for 72 h in the presence or absence of the PD-L1 blocking antibody. Consequently, no significant change in CD8^+^ T cell apoptosis and SNK-6 proliferation with or without PD-L1 blocking antibody was identified. However, a significant inhibitory effect of PD-L1 for allogeneic CD8^+^ T-cytokine secretion (IL-2 and IFN-γ) was identified and this inhibitory effect was restored with the PD-L1 blocking antibody. The results of the current study showed that PD-L1 expression in SNK-6 cells inhibits the Th1 cytokine secretion of CD8^+^ T cells *in vivo*. Although PD-L1 is frequently expressed in malignant cells, the regulatory mechanisms are not uniform and a number of complex signaling pathways are involved in its regulation in HL and NHL (with the exception of ENKL), such as MEK/ERK, NF-jB, PI3K/Akt, JAK/STAT and p38 MAPK (32,33). Furthermore, two major regulatory pathways of PD-1/PD-L2 are the NF κB and STAT6 pathways (18). However, the regulatory mechanisms of PD-1/PD-L in ENKL are less well known; therefore, future studies are required to delineate the role of PD-L interaction with tumor T cells *in vivo*.

Currently, bioimmunotherapy is a pivotal study field in the therapy of tumors. However, the immunotherapy of ENKL remains immature. The results of the current study revealed that PD-Ls and PD-1 are aberrantly expressed in ENKL and may prevent effective antitumor immunity *in vivo* by interacting with tumor T cells, which provides important information to reveal the cellular immune deficiency mechanism in ENKL. Future studies in animal models and patients are required to fully delineate the immune regulation functions of PD-Ls as well as molecules involved in the mediation mechanism of ENKL. Thus, the manner in which to selectively block these inhibitory molecules is likely to present an attractive approach to ENKL immunotherapy.

## Figures and Tables

**Table I:** Correlation between PD-L1 or PD-L2 protein expression in ENKL tissues and the clinicopathological parameters of the 30 ENKL patients.

		PD-L1, n			PD-L2, n		
			
Variables	Total, n	−	+	P-value	−	+	P-value
Gender							
Male	21	10	11	0.193	8	13	0.804
Female	9	2	7		3	6	
Age, years							
≥45	17	7	10	0.880	6	11	0.858
<45	13	5	8		5	8	
PS score							
≤80	15	7	8	0.456	5	10	0.705
>80	15	5	10		6	9	
IPI score							
≤2	20	11	9	0.018	8	12	0.592
>2	10	1	9		3	7	
Stage							
I+II	21	11	10	0.034	11	10	0.006
III+IV	9	1	8		0	9	
LDH							
<281	23	12	11	0.014	10	13	0.161
≥281	7	0	7		1	6	
β2-MG							
<3	20	9	11	0.429	7	13	0.789
≥3	10	3	7		4	6	
EBER							
Positive	25	9	16	0.317	8	17	0.236
Negative	5	3	2		3	2	
Ki-67, %							
<60	15	9	6	0.025	5	10	0.705
≥60	15	3	12		6	9	

PD-L, programmed death ligand; ENKL, extranodal natural killer/T-cell lymphoma, nasal type; PS, performance status; IPI, international prognostic index; LDH, lactate dehydrogenase; β2-MG, β2-microglobulin; EBER, Epstein-Barr virus-encoded small RNA.
